# Diagnostic Pitfall: Prolonged COVID-19 Presenting as Organising Pneumonia in a Rituximab-Treated Patient with Rheumatoid Arthritis

**DOI:** 10.31138/mjr.090326.rpr

**Published:** 2026-06-12

**Authors:** Antigoni Soufla, George E. Fragoulis, Alexios Iliopoulos

**Affiliations:** 1 Department of Rheumatology, 417 Army Share Fund Hospital (NIMTS), Athens, Greece;; 2 First Department of Propaedeutic and Internal Medicine, National and Kapodistrian University of Athens, Athens, Greece

**Keywords:** rheumatoid arthritis, autoimmune diseases, gastrointestinal microbiome, immune system, inflammation, microbiota

## Abstract

Rituximab (RTX)–induced B-cell depletion is associated with prolonged SARS-CoV-2 infection. We describe a case of persistent COVID-19 in a patient with rheumatoid arthritis (RA) initially misdiagnosed as cryptogenic organising pneumonia (COP), highlighting the diagnostic and management challenges in RTX-treated patients. Retreatment with a second 5-day course of nirmatrelvir/ritonavir led to rapid clinical improvement and complete radiological resolution. Persistent SARS-CoV-2 infection should be considered in RTX-treated patients with non-resolving pulmonary infiltrates, particularly when corticosteroid therapy is ineffective.

## INTRODUCTION

Rituximab (RTX)-induced B-cell depletion is sometimes associated with prolonged SARS-CoV-2 infection. We report a case of persistent COVID-19 in a patient with rheumatoid arthritis (RA), which was initially misdiagnosed as cryptogenic organising pneumonia (COP). Although RTX has been linked to severe and prolonged COVID-19, evidence in rheumatology cohorts is limited compared to hematologic populations. In the Omicron-era, real-world data continue to show increased COVID-19-related hospitalisations in RTX-treated patients despite vaccination and antiviral availability.^1^

Herein, we report a 56-year-old woman with seropositive, erosive RA (diagnosed in 1994) in sustained remission on two doses of rituximab 1000 mg every six months since 2018. She had previously been treated with multiple DMARDs/biologics and had a history of multiple orthopaedic surgeries.

## CASE PRESENTATION

In September 2024, one day after her first scheduled RTX infusion she developed fever, malaise and dry cough; SARS-CoV-2 infection was confirmed by rapid antigen testing. This was her second COVID-19 episode despite four doses of the BNT162b2 (Pfizer-BioNTech) vaccine (last dose 8 months earlier). She completed a 5-day course of nirmatrelvir/ritonavir with initial clinical recovery, and the second RTX dose was administered as scheduled.

**Figure 1.**
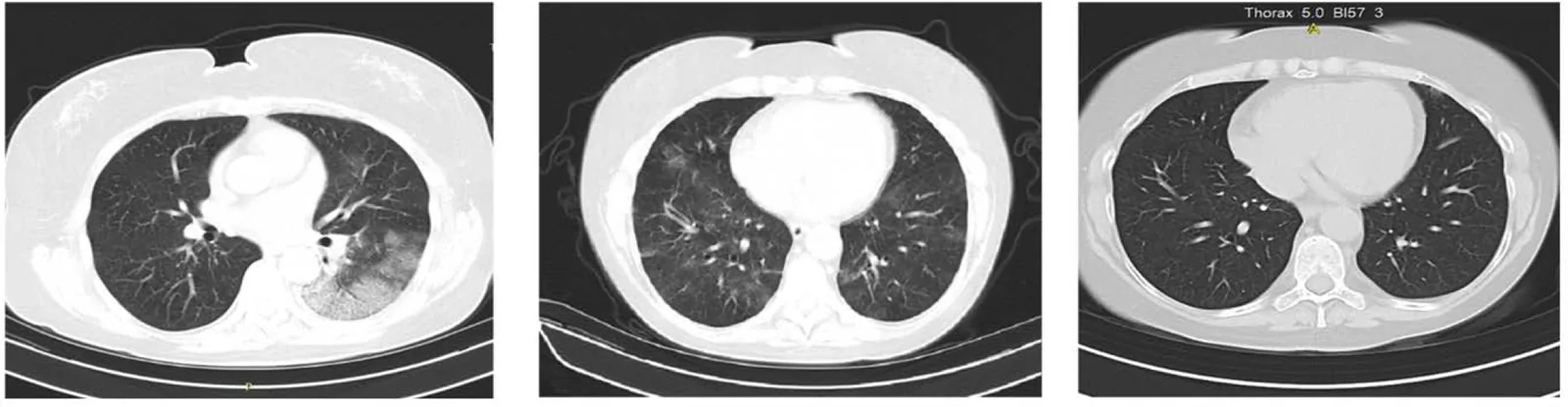
Serial chest CTs: (**A**) January 2025 (predominant left lower-lobe consolidation (**B**) April 2025 (diffuse ground glass opacities), (**C**) June 2025 (complete resolution).

Approximately 15 days later, she developed a progressively worsening dry cough, followed by dyspnoea one month later, and she represented in January 2025 with fever and exertional dyspnoea. Chest CT (**Figure 1A**) revealed bilateral consolidations and ground-glass opacities, predominant in the left lower and right upper lobes. Empirical moxifloxacin and azithromycin were ineffective, and she was hospitalised. Blood cultures; rapid antigen tests for SARS-CoV-2, RSV, and influenza; nasopharyngeal SARS-COV-2 PCR; sputum cultures; and bronchoalveolar lavage (BAL) with microbiological and PCR analyses, including Mycobacterium tuberculosis, were all negative. Based on imaging and lavage findings, she was treated for presumed COP with ceftriaxone and high-dose corticosteroids (methylprednisolone 80mg daily, tapered to 60mg in hospital and 40mg at discharge).

Despite treatment, respiratory symptoms persisted and ESR, CRP remained elevated. A repeat CT in April showed diffuse ground-glass opacities and scattered consolidations (**Figure 1B**). Although antigen tests remained negative, two independent PCR assays were positive for SARS-CoV-2, confirming persistent infection. A second 5-day course of nirmatrelvir/ritonavir led to rapid symptomatic improvement, and follow-up CT two months later (**Figure 1C**) demonstrated complete radiologic resolution.

## DISCUSSION

This case highlights the diagnostic and therapeutic challenges of COVID-19 in patients treated with rituximab. First, diagnostic delay is common. Rituximab-induced B-cell depletion markedly impairs viral clearance, leading to prolonged SARS-CoV-2 replication. Importantly, in this context, nasopharyngeal sampling has reduced sensitivity. A multicentre cohort study reported nasopharyngeal PCR positivity in only 52.2% of confirmed cases, compared with 70.6% positivity in bronchoalveolar lavage samples, which may explain the initially negative upper-respiratory testing observed in our patient.^2^

Second, immunological vulnerability plays a central role. Patients treated with rituximab are unable to mount effective humoral responses, even after mRNA vaccination: 29% develop neutralising antibodies, compared with around 80% of patients receiving conventional immunosuppressive therapies. This humoral immunodeficiency-often persisting for 6–12 months after rituximab administration-impairs both natural immune recovery and vaccine-induced protection, thus patient is vulnerable to prolonged SARS-CoV-2 infection.^3^

Anti-CD20 therapy predisposes to serious pulmonary complications when combined with COVID-19 infection. In this setting, COVID-19–associated secondary organising pneumonia has been increasingly reported in B-cell-depleted patients, with biopsy-proven cases documented in multiple sclerosis cohorts treated with ocrelizumab and rituximab, occurring weeks to months after SARS-CoV-2 infection. By contrast, in our patient, persistent COVID-19 mimicked COP radiologically but resolved completely with antiviral therapy, without histologic confirmation of organising pneumonia.^4^

Concerns have also been raised regarding viral rebound following antiviral therapy. In a retrospective study of 15 913 adults treated with nirmatrelvir/ritonavir, risks of both virologic and symptomatic rebound were higher during the Omicron BA.5-predominant period compared with the BA.2.12.1-predominant period (HR 1.32, 95% CI 1.06–1.66 and HR 1.32, 95% CI 1.04–1.68, respectively).^5^ These findings suggest that increasing immune evasiveness of successive variants may contribute to rebound risk and support evaluation of extended or repeated antiviral treatment in selected patients. Consistent with this approach, Shimizu et al. reported successful treatment of persistent SARSCoV-2 infection in a rituximab-treated patient with nephrotic syndrome using a combination of remdesivir and monoclonal antibodies.^6^ Rituximab-associated persistent COVID-19 has been well documented in haematologic immunosuppression, where it is associated with increased mortality.^7^ Similar patterns of prolonged infection with persistent or migratory pulmonary infiltrates and repeated lower-respiratory PCR positivity have also been reported in rituximab-treated patients with multiple sclerosis, underscoring the broader relevance of anti-CD20-associated viral persistence.^8^

Clinically, persistent or recurrent respiratory symptoms following COVID-19 in RTX-treated patients should prompt suspicion of ongoing viral replication rather than post-infectious or autoimmune lung disease. Repeated testing, including lower-respiratory sampling, may be required to establish or exclude infection. Early recognition and retreatment with antiviral therapy can lead to rapid recovery and may prevent unnecessary escalation of immunosuppression and prolonged morbidity.

## KEY MESSAGE

Rituximab-associated B-cell depletion predisposes to persistent COVID-19 mimicking organising pneumonia.
